# Retraction Note: LINC00115 promotes stemness and inhibits apoptosis of ovarian cancer stem cells by upregulating SOX9 and inhibiting the Wnt/β-catenin pathway through competitively binding to microRNA-30a

**DOI:** 10.1186/s12935-024-03561-5

**Published:** 2024-11-08

**Authors:** Rui Hou, Luo Jiang

**Affiliations:** 1 Department of Obstetrics and Gynecology, Shengjing Hospital of China Medical University, Shenyang, PR China; 2 Department of Ultrasound, Shengjing Hospital of China Medical University, 36 Sanhao Street, Shenyang, 110004 PR China

**Retraction Note: Cancer Cell International (2021) 21:360**

10.1186/s12935-021-02019-2.

The Editors-in-Chief have retracted this article. After publication, concerns were raised regarding high similarity between Fig. 8D sh2 image in this article and Fig. 7C MCF-7/Tax si-LINC00160 in [1]. Additionally, the western blot images in this article appear similar to those in a number of other articles on non-coding RNAs published within a similar time frame.

The authors have stated that the images in Fig. 8D were provided by a third party, and were unable to provide the original western blot scan files for validation.

The Editors-in-Chief therefore no longer have confidence in the presented data.

Luo Jiang has stated on behalf of both authors that they agree to this retraction.
